# Over-Stretching Tolerant Conductors on Rubber Films by Inkjet-Printing Silver Nanoparticles for Wearables

**DOI:** 10.3390/polym10121413

**Published:** 2018-12-19

**Authors:** Andreas Albrecht, Marco Bobinger, José F. Salmerón, Markus Becherer, Gordon Cheng, Paolo Lugli, Almudena Rivadeneyra

**Affiliations:** 1 Institute for Nanoelectronics, Technical University of Munich, Theresienstr. 90, München 80333, Germany; andreas.albrecht@tum.de (A.A.); marco.bobinger@tum.de (M.B.); jf.salmeron@tum.de (J.F.S.); markus.becherer@tum.de (M.B.); 2 Institute for Cognitive Systems, Technical University of Munich, Karlstr. 45, München 80333, Germany; gordon@tum.de; 3 Free University of Bozen-Bolzano, Universitätsplatz 1, Bozen-Bolzano 39100, Italy; paolo.lugli@unibz.it; 4 Pervasive Electronics Advanced Research Laboratory (PEARL), Department of Electronics and Computer Technology, University of Granada, 18071 Granada, Spain

**Keywords:** inkjet printing, printed electronics, silver nanoparticles, stretchable, wearables

## Abstract

The necessity to place sensors far away from the processing unit in smart clothes or artificial skins for robots may require conductive wirings on stretchable materials at very low-cost. In this work, we present an easy method to produce wires using only commercially available materials. A consumer grade inkjet printer was used to print a wire of silver nanoparticles with a sheet resistance below 1 Ω/sq. on a non-pre-strained sheet of elastic silicone. This wire was stretched more than 10,000 times and was still conductive afterwards. The viscoelastic behavior of the substrate results in a temporarily increased resistance that decreases to almost the original value. After over-stretching, the wire is conductive within less than a second. We analyze the swelling of the silicone due to the ink’s solvent and the nanoparticle film on top by microscope and SEM images. Finally, a 60 mm long stretchable conductor was integrated onto wearables, and showed that it can bear strains of up to 300% and recover to a conductivity that allows the operation of an assembled LED assembled at only 1.8 V. These self-healing wires can serve as wiring and binary strain or pressure sensors in sportswear, compression underwear, and in robotic applications.

## 1. Introduction

The opportunities to improve processes and healthcare by using more and more sensors in the system or on the human body increase the need for sensors on a large range of surfaces. These sensors need electrical wiring, which may be realized on stretchable materials like textiles and rubbers [1]. Electronics on stretchable materials are required for smart clothing in healthcare and sports, implants integrated within the body, as well as stretchable sensor skins for humanoid robots and objects with high mechanical strain.

In recent years, researchers utilized two complementary ways to achieve conductive patterns on stretchable materials. The first method applies conventional materials like gold, silver and other metals that are sufficiently thin to be bendable onto a sheet of stretchable material like rubber [2,3,4,5,6,7]. The second approach uses new materials that are intrinsically stretchable or able to rearrange their components inside a stretchable matrix to realize flat patterns [8]. Among them are materials based on metal particles [9,10,11] or wires [12], conductive polymers [12,13], graphene [14,15], carbon nanotubes [16] and hybrid structures [17,18]. Both strategies have distinct advantages and disadvantages: The former offers the high conductivities of metals and semiconducting properties of thin silicon sheets and can be produced with conventional semiconductor technology. However, vacuum processes, the requirement for a very planar surface, and the low abrasion resistance of the layers make them not easily usable for wearables. The latter utilizes much cheaper processes and materials that are already fulfilling the requirements as a bulk material [19]. Additive manufacturing techniques like inkjet or screen printing can be utilized to apply such materials. On the other side, common resistances are rather high [20].

This work aims to find a compromise between both approaches. We show highly conductive metal thin films that can be produced by low-cost inkjet-printing on a stretchable material. We focus on one widely used polymer, PDMS, a silicone rubber with high elasticity, optical transparency, low-cost, and easy processing. PDMS is viscoelastic, meaning that the strain is not only force-dependent but also time-dependent. PDMS is almost incompressible, having a Poisson ratio of close to 0.5, meaning that every compression or strain in one dimension leads to an opposite variation of at least one other dimension [21].

The conductivity of the wires in this work is achieved by silver nanoparticles (AgNP). In contrast to most new materials like carbon black, carbon nanotubes, graphene-flakes, PEDOT:PSS, and other conductive polymers, the conductivity of metals is some orders of magnitude higher [19,22]. AgNPs with diameters below 100 nm can be dispersed with suitable capping agents in solvents and then inkjet-printed like colour ink [23]. If these capping agents are well-designed, they degrade over time at room temperature or slightly elevated temperature and the thin-film is conductive without other forms of post-treatment [22,23,24].

Printing technologies promise a much simpler and more environmentally-friendly production process at environmental conditions. In particular, inkjet-printing is a very versatile printing technique that is especially suitable for printing on rubbers. It is a contact-less printing technique and does not exert pressure on the plain substrate and on previously printed layers on the substrate [25,26].

## 2. Materials and Methods 

### 2.1. Materials and Printing Equipment

The AgNP ink DGP-40LT-15C (Advanced Nano Products (ANP), Sejong-si, Korea) was used without either chemical modification or processing except for shaking by hand prior to filling the cartridge. The used PDMS film was an Elastosil film (Wacker Chemie AG, Munich, Germany) that was oxygen plasma treated for 60 s at about 100 W generator power and 0.3 mbar oxygen pressure with a Femto Plasma Asher (Diener Electronics, Ebhausen, Germany). The ink was printed within 30 min after the plasma treatment onto the silicone film by a low-cost consumer inkjet printer, Epson Workforce 2010W (Memmert GmbH + Co.KG, Schwabach, Germany). The printer was used without further modification except the replacement of the black cartridge by a refillable cartridge filled with the AgNP ink. After printing, the samples were dried at 60 °C for 30 min in an oven (Memmert UF 55, Memmert GmbH + Co.KG, Schwabach, Germany).

### 2.2. Characterization

To evaluate the wetting behavior, we designed and printed short 100 µm wide lines. For the stretching experiments, we chose 2 mm wide and 60 mm long wires terminated by 5 × 5 mm² contact pads. The pads were only partially clamped into the characterization setup to reduce damage to the silver layer by abrasion on contact with the clamps.

Optical microscope images were taken with an Axiolab A1 MAT equipped with an Axiocam 105 color camera (both of Carl Zeiss AG, Oberkochen, Germany). Profilometer studies were conducted with a Dektak XT (Bruker Corporation, Billerica, MA, USA). Scanning electron microscope (SEM) images were taken with a Nvision 40 (Carl Zeiss AG, Oberkochen, Germany) at 2.0 kV beam energy and a magnification of 10,000.

**Figure 1:**
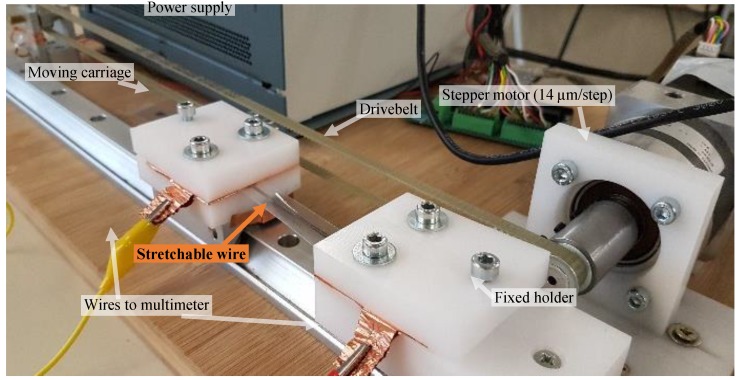
Characterization setup with a stretchable wire between the moving carriage and the fixed holder.

The strain to the printed conductor was exerted with a self-built setup shown in Figure 1 including a stepper motor (Nanotec Electronic GmbH & Co KG, Feldkirchen, Germany) with a step length of 14 µm. The samples were mounted between a fixed holder and a moving carriage on a linear profile. Both mounting positions were coated with a copper tape to minimize the contact resistance by setting up a large contact area. The distance between them was measured before the experiment. The moving carriage was moved 10,000 times to stretch the sample by 20% and then relax it to the original position. The speed in both directions was adjusted, so that the carriage was moving for 1 s. The two-point-resistance was measured with a Keithley 2700 Multimeter (Keithley Instruments, Cleveland, OH, USA). A LabView program automated the process by measuring the resistance about 10 s after the relaxation of the sample and then triggering a new cycle. The sheet resistance was calculated neglecting contact resistances with the measured line widths and lengths.

### 2.3. Integration into Wearable

Sylgard 184 PDMS purchased at Sigma Aldrich (St. Louis, MO, USA) was used to glue the silicone film with the conductive onto regular women tights and to mechanically stabilize the connection wires onto the film. The PDMS was cured for 30 min at 100 °C in an oven. The electrical connection and the assembly of the light emitting diode were made with conductive epoxy. Both materials were cured at 90 °C for 30 min. A voltage of 1.8 V was applied to power the LED with a regular power supply.

## 3. Results

### 3.1. Print Quality Assessment of Ag Patterns

Only after exposing the used silicone film to oxygen plasma, it was possible to wet the substrate sufficiently to achieve continuous lines. We found that a very wide range of parameters (power, time, and pressure) for the plasma treatment were able to achieve the desired wettability, however, this could possibly influence the process time window before inkjet printing. Figure S1 in the Supplementary Information shows a comparison of printed areas, vertical and horizontal lines on untreated silicone films (a–c) and plasma-treated silicone films (d–f). The satellite drops near the vertical lines are resulting from inaccuracies of the cheap desktop inkjet printer [23]. The printing time of a few seconds is much shorter than the drying time of the ink, so the ink drops can merge together to achieve a homogeneous area coverage. This shows the advantage of the use of a consumer inkjet printer in comparison to many groups that employed slow lab-scale inkjet printers. We tried to repeat the results with a dedicated inkjet printer (Dimatix DMP-2850 from Fujifilm Dimatix Inc., Santa Clara, CA, USA), but were not able to overcome the formation of printed line patterns within the wires.

To obtain conductive lines, there is a minimum line-width for two substrate-related reasons: thin lines have a very small amount of ink and every small defect of the substrate can cause an error in the conductive path. Some of these very small and local defects can be seen in Figure S1f as dark spots on and next to the printed silver line. More important, however, is the occurrence of changes in the wettability of the substrate. On some areas of several tens of mm², the substrate appears to be more hydrophobic than on the rest. We selected a linewidth of 1–2 mm as width to ensure the reliability of stretchable inkjet-printed conductors. It must be highlighted that the sequence of drop matrix formation and landing (the number of drops generated simultaneously together with its timing and ink volume) could possibly influence the “drop merging” and therefore, the lines conductivity [20]. In our concrete case, the nozzle plate of the inkjet printer has two rows of nozzles separated about 3 cm, containing 180 nozzles each of 25 µm diameter, and being the distance between nozzles 141 µm [23].

**Figure 2:**
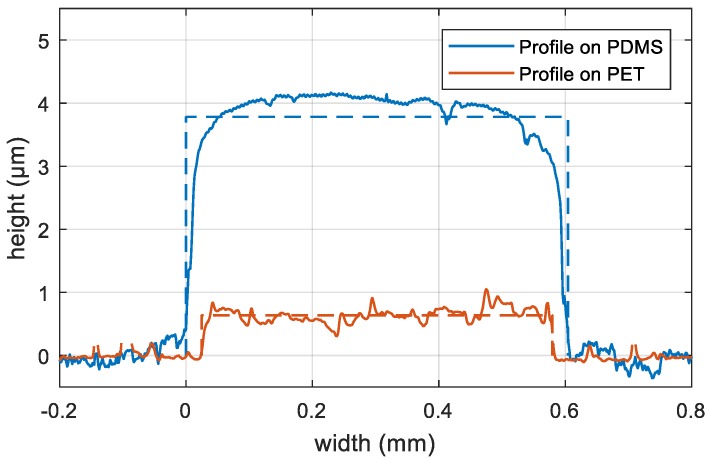
Height profile of inkjet-printed silver lines on PDMS and PET. Horizontal dashed lines show the average height of 3.8 µm and 0.6 µm, respectively, calculated over the line width marked by vertical dashed lines.

Figure 2 shows the profilometer measurement of two inkjet-printed silver films on PDMS and polyethylene terephthalate (PET), respectively that were printed with the same settings on the same printer. The thickness measurement of the inkjet-printed silver film shows a substantially higher film thickness than on other substrates. On PET and other substrates, e.g., paper, the thickness ranges between 0.6 and 0.8 µm, whereas the lines on PDMS are 3.8 µm thick on average. The measured value can originate only partially from the silver layer because the inkjet printer applies the same amount of ink on any substrate. The major part of the height may be caused by swelling of PDMS after solvent absorption. A useful parameter for an assumption is the Hansen total solubility parameter [27,28,29]. For TGME, the solvent of the AgNP ink, this parameter can be calculated as 21.8 (J/cm³)^0.5^ using the parameters for TGME of Hansen [30]. Solvents having a similar parameter (±5%) show a swelling of 3% to 21% in the study of Lee et al. [29] The observed difference in height of 3.1 µm is reached, if we assume a value in the middle of this interval and the solvent penetrates the top 15 µm of the PDMS substrate.

### 3.2. Electro-Mechanical Characterization

**Figure 3:**
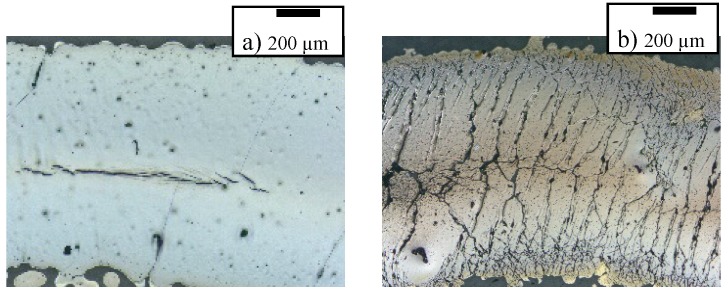
Inkjet-printed line before (**a**) and after (**b**) stretching 2000 times. After stretching, many small cracks occur within the printed surface.

The original resistance of the 2 mm wide and 65 mm inkjet-printed silver wire was 23.7 Ω, resulting in a sheet resistance of 730 ± 50 mΩ/sq. Assuming a silver layer thickness of 600 nm, the conductivity is about 1.82 × 10^4^ S/cm. This is far below bulk metal layers (>10^7^ S/cm) but much higher than for most composite materials (10^-5^ to 100 S/cm) [31,32]. Figure 3 shows comparative optical microscope images of the wires before and after stretching of 20% for 2000 times. Before stretching the 2 mm wide wires, only a few cracks are visible. These may be caused by the relaxation of the rubber film after thermal expansion during drying. After stretching, more and more cracks appear that form a widely interconnected network. On most observed wires, the cracks in the center appeared to be more pronounced than the ones on the edges; although the density of cracks per unit area is higher on the edges.

**Figure 4:**
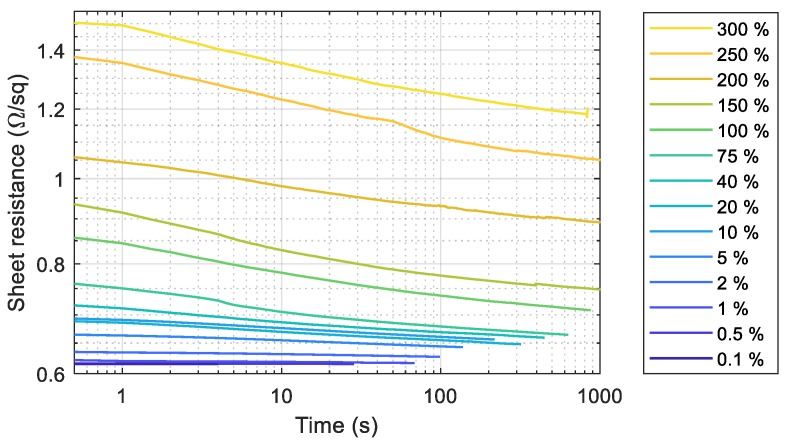
The sheet resistance of the inkjet-printed silver wires after stretching cycles that consist of a stretching of 20% that breaks the conductive paths in the wire and a relaxation after a hold-time of 10 s.

The silver wire was exposed to different strain on our custom-made stretching setup (Figure 1) and then relaxed to the original position with a speed of about 60%/s. We started at small strains of 0.1% and increased it gradually until the breaking point, which is between 300% and 350%. In the relaxed position, we measured the recovery of the resistance after 0.5 s, after 30 s and after longer periods of time. Figure 4 shows the recovery rates of the different strains. Small to medium strains of less than 80% only lead to a small increase of the resistance of less than 0.2 Ω/sq. High strains of 100% up to 300% increase the sheet resistance by a factor of 1.5 to 2.5, which is still in the tolerance of most systems. After some time in the relaxed position, the resistance decreases logarithmically towards the original value. It must be mentioned that the measurement of the high strain values was conducted before the substrate relaxed completely. Thus, the real resistance values may be even lower than the shown ones.

**Figure 5:**
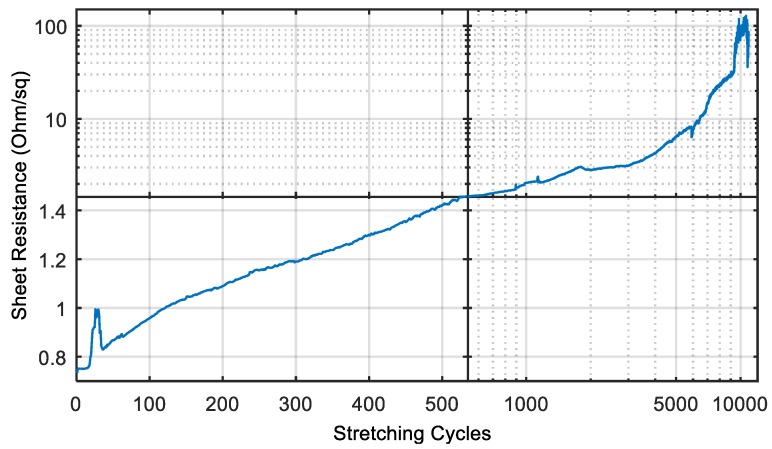
The sheet resistance of the inkjet-printed silver wires after stretching cycles that consist of a stretching of 20% that breaks the conductive paths in the wire and a relaxation after a hold-time of 10 s.

For the following experiments, we chose a strain of 20% and repeated the stretching and relaxing in cycles. The resistance was measured after 30 s in the relaxed position. The measured resistance after each cycle is shown in Figure 5. After one cycle, the resistance increased by 2.4% to 24.3 Ω. For the next 15 cycles, the resistance changes less than 1% of the last value. This surprising behavior shows that metal nanoparticle films exhibit similar electrical properties to very thin bulk metal films, e.g., 50 nm of gold in the work of Graz et al. and Jones et al. [33,34].

After 20 cycles, the resistance increased rapidly to up to 135% of the original value. This high resistance value was only measured for about 10 cycles. We found, that sometimes such peaks occur directly after relaxation. Mostly, the high conductivity is established after less than 1 s, but sometimes it takes several seconds. Thus, we conducted the measurement 10 s after relaxation and could avoid most of these peaks. After the 30^th^ cycle, the resistance dropped again and from there on, it increased steadily after each strain cycle. After 500 cycles, the resistance still not yet doubled (1.42 Ω/sq.), which is acceptable for most applications. We could stretch the sample with this measurement protocol for almost 6500 cycles before the resistance exceeded 10 Ω/sq. After 10,000 cycles, the printed wire was still conductive, however, with a high resistance of about 60 Ω/sq. (80 times the original value).

We assume that the increase in resistance is related to the viscoelastic behavior of silicones. Within the 10 s of relaxation, the film did not have enough time to relax to its original position. After 5000 cycles, the film was still stretched by 1–2% in the relaxed position. The remaining strain forms gaps between the AgNPs.

**Figure 6:**
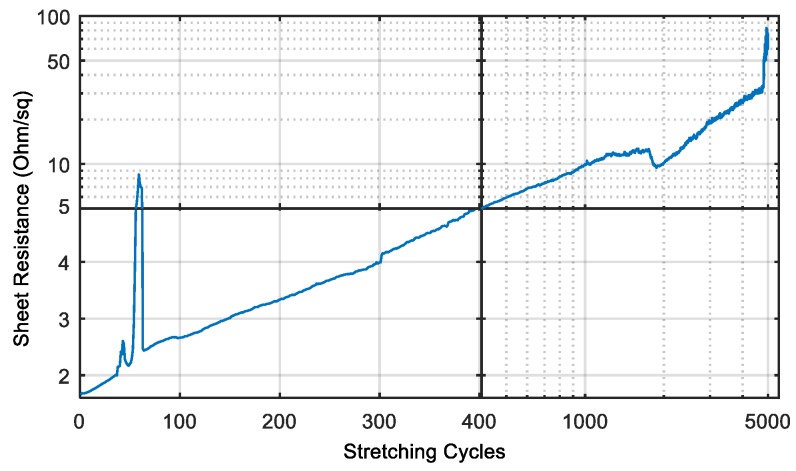
The sheet resistance of the inkjet-printed silver wires after two days of relaxation during the cyclic stretching test.

Two days later, we continued the measurement with exactly the same sample without removing it from the setup and measured the resistance again after each cycle (Figure 6). In the meantime, the resistance dropped to 55 Ω or 1.66 Ω/sq. while keeping the wire in the relaxed position without any treatment or processing. This behavior can also be explained by the viscoelastic behavior of PDMS. The Elastosil film requires a couple of hours to form back to the original state. According to the manufacturer, the film relaxes back to 100% of its original length after 24 h.

### 3.3. Reasons for the Self-Healing

**Figure 7:**
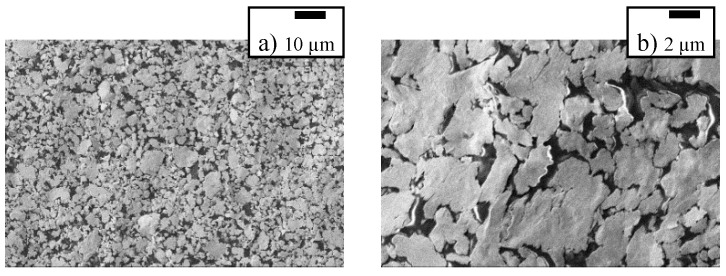
SEM image of a cracked silver nanoparticle film showing many micrometer-scaled silver flakes that can form a conductive path while relaxed: (**a**) scale of 10 µm and (**b**) zoom at 2 µm.

**Figure 8:**
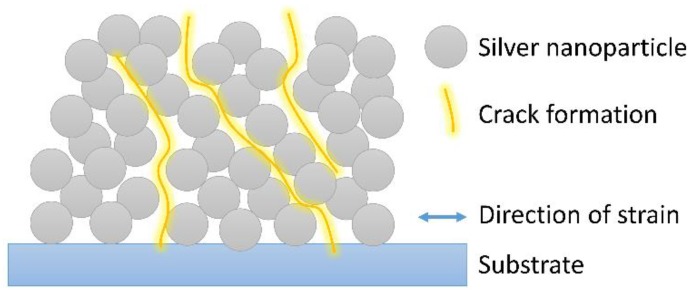
Schematic of the crack formation within the silver nanoparticle film. The schematic may be applied in the *x*-*y* plane and in the *x*-*z*-plane. All cracks lead to a lattice deformation that either preserves conductive paths or moves the lattice so that a conductive path is restored after relaxation.

The investigation of the local behavior was conducted on an SEM. The SEM images in Figure 7 shows that the unsintered metal film offers a high number of possible crack locations that are predefined by the nanoparticle structure. The attractive forces between unsintered nanoparticles are weak and crack formation between them are very likely. The absence of a lattice like in bulk metals allows very long and complex cracks to develop. We assume that are only very few cracks in the nanoparticle film, that completely interrupt the current flow. Many cracks only partially cross the film and release strain. This behavior occurs in the plane of the film but also may occur through the z-axis of the film. Figure 8 shows a schematic of a section of the nanoparticle film with one crack through the entire film and two that are not interrupting the current flow. A film thickness of about 600 nm consists only of about 10–15 nanoparticles on top of each other. The schematic only shows about half of the thickness.

The formation of a crack always locally moves the lattice of the nanoparticles, so that there are still a few nanoparticles that are conducting current within the cracks. Although they are not connected during the stretched state, they form a well-conducting connection in the relaxed state. To further enhance this self-healing effect different approaches could be followed, like increasing the thickness of printed wires or optimizing the wire layout.

### 3.4. Implementation on a Wearable

Self-healing stretchable conductors are essential for the application in wearables. Clothes are exposed to a large strain when they are put on and taken off. In particular, this property is important for all clothes that should lie flat on the skin, e.g., compression wear and sensing underwear. During dressing and undressing, the functionality of the embedded electronics is not necessary. Pulse measurements, sweat analysis, electrocardiography, pressure measurements and many others would produce erroneous measurements. We tested our conductive wires for stretch cycles up to 300% of strain. At a strain of more than 330%, the substrate of most of our test samples broke. Typical elongations in sportswear and compression wear are less than 100% and much lower than the limitation of the Elastosil film [12,35].

**Figure 9:**
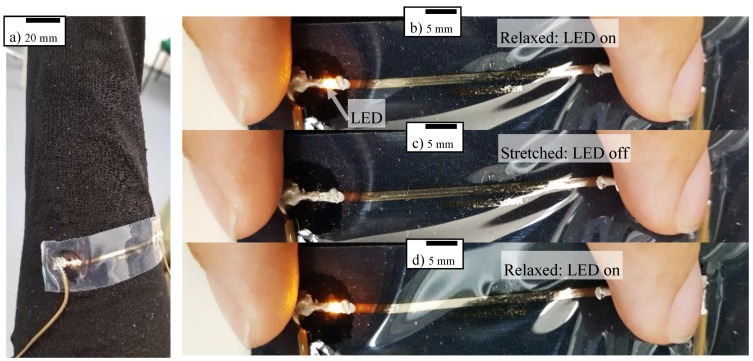
(**a**) Stretchable wire attached to tights with connecting wires and a light emitting diode (LED). The LED is lighting in the relaxed position (**b**), not lighting in a stretched state (**c**), and again lighting when returning to the relaxed position (**d**).

Figure 9a shows one of our stretchable wires attached to regular women’s tights. Two connection wires (brown) were glued onto the pads at the end of the wire. A part of the wire close to the end was scratched off and a light emitting diode (LED) was placed across the gap. This shows that – in principle – other components can be assembled to the film as well. A voltage of 1.8 V made the LED light up brightly in the relaxed state. Figure 9b–d show one manual strain cycle starting in the relaxed position with the lighting LED. When stretching the wire slightly, the connection is lost and the LED fades out. Releasing the strain, this is reversed and the LED lights up again. A video of several cycles can be found in the Supplementary Information (Video S1). Such a product can be used as a sensor to monitor the strain of the tight as well as the pressure onto the leg, two important criteria for compression wear and body-shaping underwear. Such sensors can survive large strains that occur during dressing and at certain parts of the body, e.g., knee or heel.

### 3.5. Discussion

In this section, we briefly recap the state-of-the-art for stretchable conductors and compare the recent findings in the literature with our devices. The relevant device and manufacturing properties such as the i) electrode material, ii) deposition technique, iii) substrate material, iv) resistivity, v) thickness, vi) treatment, vii) strain and viii) the increase in resistance with respect to the relaxed state of the device, are summarized in Table 1. As outlined in the introduction section and summarized in Table 1, a large variety of electrode materials including AgNPs [36,37,38,39], silver flakes [40], CNTs [40], PEDOT:PSS [41] and AgNWs [42,43] have been reported for stretchable conductors. These materials were deposited by extremely diverging techniques such as direct printing with a single nozzle [36], inkjetting [37], stamping using a PDMS-based master [38], electrospinning of a fiber mat [39], as well as simple drop casting [40,42,43] and spin coating [41]. In order to promote the conductivity, following treatments have been reported: i) no treatment besides the curing of the PDMS-substrate at a temperature of 65 °C for a duration of 12 h [42], ii) annealing under ambient conditions in a temperature range of 100 °C–250 °C for a duration of 5 min–60 min [36,37,38,40,41], iii) ultraviolet (UV) light curing [43] and iv) reduction of the electrospun fiber mat in a silver-containing precursor to synthesize the nanoparticles [39]. When compared to the aforementioned protocols and materials, it can be concluded that our stretchable conductors exhibit a high conductivity and can be strained to around 300% of the initial length, at a moderate increase in resistance of a factor of around 75. Besides these good performance parameters, the electrodes presented in this study can be deposited with a low-cost inkjetting technique and the as-deposited films only require a mild post-processing temperature of 60 °C, for a duration of 30 min. However, an important but commonly overseen aspect for stretchable conductors is their adhesion to the substrate, which will be studied in the following. To study the adhesion of the silver lines to PDMS, standard 3M scotch tape was utilized. It could be observed (see supporting video 1) that the traces were peeled off after the first adhesion test. This is an expected result for non-protected nanostructures because adhesion is a common problem [44] but it can be easily enhanced by protecting the printed silver lines using a thin coating layer (i.e., PMMA or sputtering of evaporating oxides) on top of it [45]. To retain the stretchability of the conducting material, the silver lines can also be sandwiched in PDMS using a PDMS-based encapsulation [45].

## 4. Conclusions

We showed that plasma treated PDMS films can serve as a substrate for inkjet-printed silver. The inkjet-printed silver film is almost crack-free after drying at 60 °C and shows a sheet-resistance below 1 Ω/sq. without any sintering step. The irreversible swelling of PDMS after contact with certain solvents did not inhibit the use of the printed wires. The wires printed for this work are stretchable up to a few percent. During cyclic stretching and relaxing of the wires, we found that the resistance only slightly increases during the first few cycles. After almost 6500 cycles with only 10 s relax time, the sheet resistance is still below 10 Ω/sq. If the wires are left untouched for more than 24 h, the resistance was reduced to about twice the original value, although stretched for more than 10,000 cycles. The increasing resistance during our stretching test and the recovery after a longer pause can be explained by the viscoelastic nature of PDMS. During stretching, the film viscoelastically elongates by a few percent and requires a relaxation time longer than our cycle length to reach its original length. When the film recovers to its original length, most conductive paths are restored and the resistance recovers.

Our stretchable wires possess a high stretchability of more than 300% and thus are suitable for applications on most stretchable textiles used for wearables. We showed the integration onto wearables, e.g., highly elastic tights, together with the assembly of components, e.g., an LED. When the substrate is in the relaxed position, the LED lights up. On excess strain or pressure onto the part of the body below, the electrical connection is open, and the LED turns off. This may find use in compression wear and body-shaping underwear, which aim for a homogeneous and specific strain and pressure distribution. Furthermore, the over-stretching tolerant wires may find use in an artificial skin for humanoids.

## Figures and Tables

**Table 1:** Summary for the manufacturing and material properties of stretchable conductors including this work.

Ref.	Electrode	Method	Substrate	Resistivity [10^−6^∙Ω∙cm]	Thickness [µm]	Treatment	Strain	*R*/*R*_0_; #cycles
[36]	AgNP	Printing	polyimide	52	N.A.	250 °C, 30 min	10%	Stable; #200
[37]	AgNP	Inkjetting	PDMS	71	1.6	100 °C, 1 h	10%	3; #1000
[38]	AgNP	Stamping	PDMS and PET	6.7 × 10^3^	N.A.	160 °C, 5 min	7%	Stable; #1
[39]	AgNP/rubber fibres	Electrospinning	Fiber mat	1.8∙× 10^2^	150	Reduction	40%	Stable; #300
[42]	AgNW	Drop casting	PDMS	1.9 × 10^2^	3	PDMS cur.	50%	Stable; #50
[43]	AgNW/poly(acrylate)	Drop casting	Glass	1.3∙× 10^5^	170	UV light	20%	2.1; #600
[40]	Ag/MWCNTs	Drop casting	(NBR)	1.8∙× 10^2^	140	160 °C	20%	3.8; #5000
[41]	PEDOT:PSS	Spin coating	PDMS	46 Ω/sq.	N.A.	120 °C, 5 min	10%	Stable; #5000
This work	AgNP	Inkjetting	PDMS	55	0.6	60 °C, 30 min	300%	Stable; #10000

## Supplementary Materials

Figure S1: Inkjet-printed silver patterns on untreated silicone (**a–c**) and plasma-treated silicone (**d–f**). (**a**,**d**): printed areas larger than the image area, (**b**,**e**): printed vertical lines, (**c**,**f**): printed horizontal lines. The satellite drops in picture b and e result from inaccuracies of the cheap desktop inkjet printer [23]. Video S1: Implementation on a wearable, Video S1.
