# Supplementary material for: Over-Stretching Tolerant Conductors on Rubber Films by Inkjet-Printing Silver Nanoparticles for Wearables
Source: Polymers (Basel). 2018 Dec 19;10(12):1413. doi: 10.3390/polym10121413 (PMC6401758; doi:10.3390/polym10121413)
Supplement: Supplementary file 1 [file polymers-10-01413-s001.pdf]

Supplementary Materials: Over-Stretching Tolerant Conductors on Rubber Films by Inkjet-Printing Silver Nanoparticles for Wearables

Andreas Albrecht, Marco Bobinger, José F. Salmerón, Markus Becherer, Gordon Cheng, Paolo Lugli and Almudena Rivedeneyra

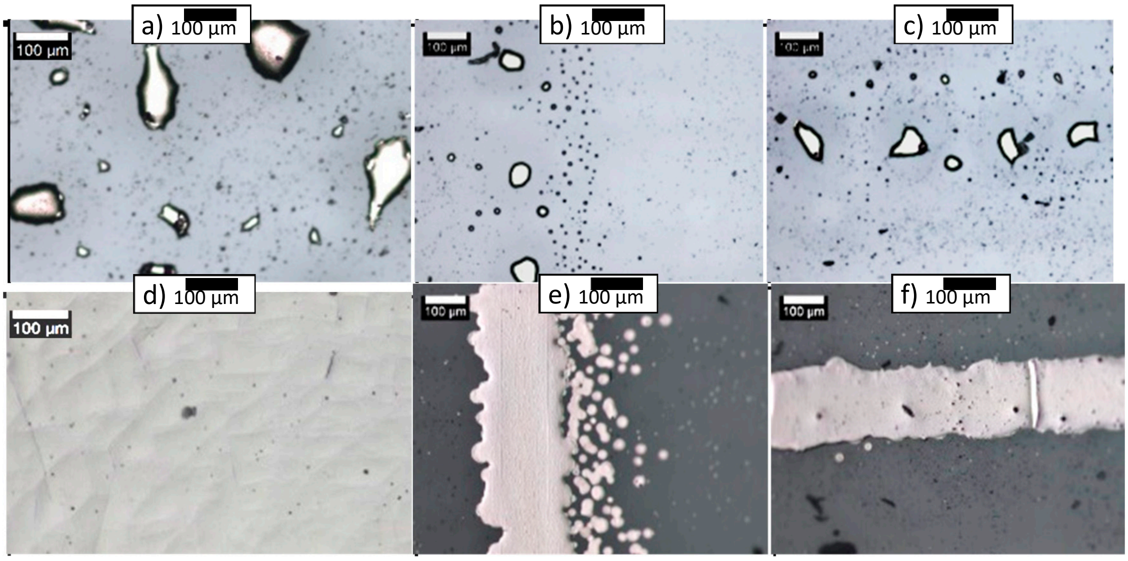

Figure S1. Inkjet-printed silver patterns on untreated silicone (**a**–**c**) and plasma-treated silicone (**d**–**f**). (a,d): printed areas larger than the image area, (b,e): printed vertical lines, (c,f): printed horizontal lines. The satellite drops in picture b and e result from inaccuracies of the cheap desktop inkjet printer [23].
